# EasyNAT MTC assay: A simple, rapid, and low-cost cross-priming amplification method for the detection of mycobacterium tuberculosis suitable for point-of-care testing

**DOI:** 10.1080/22221751.2021.1959271

**Published:** 2021-08-01

**Authors:** Zhuman Zhang, Jian Du, Tao Liu, Fen Wang, Junnan Jia, Lingling Dong, Liping Zhao, Yi Xue, Guanglu Jiang, Xia Yu, Hairong Huang

**Affiliations:** 1 National Clinical Laboratory on Tuberculosis, Beijing Key Laboratory for Drug Resistant Tuberculosis Research, Beijing Chest Hospital, Capital Medical University, Beijing Tuberculosis and Thoracic Tumor Institute, Beijing, People’s Republic of China; 2 Department of Anesthesiology, Beijing Chest Hospital, Capital Medical University, Beijing Tuberculosis and Thoracic Tumor Institute, Beijing, People’s Republic of China

**Keywords:** Tuberculosis, diagnosis, Ustar EasyNAT MTC assay, Xpert MTB/RIF, point of care testing

## Abstract

More sensitive, rapid, and affordable diagnostic tools for pulmonary tuberculosis (PTB) are urgently needed. This study aimed to assess the performance of EasyNAT MTC (abbreviation: EasyNAT) (Ustar Biotechnologies, China), a novel isothermal amplification method with a turnaround time of less than two hours that requires a few manual steps to process the sputum. Sputum samples from 249 patients with suspected PTB were subjected to smear, culture, Xpert MTB/RIF (Cepheid, USA) and EasyNAT assay testing. Of the 169 PTB patients, EasyNAT detected more PTB patients than Xpert (72.19% vs. 61.54%, *P *< 0.05, χ^2 ^= 4.326). Both the Xpert assay and EasyNAT assay detected almost all the culture-positive sputa successfully, but EasyNAT yielded more positive results among the smear-negative and culture-negative PTB cases (44.59% (33/74) vs. 22.97% (17/74), *P *< 0.01, χ^2 ^= 7.732). Although the specificity of EasyNAT was lower in contrast to Xpert [95.00% (76/80) vs. 98.75% (79/80)], the difference was not significant (*P* = 0.363, χ^2 ^= 0.826). EasyNAT could be used as an initial test for PTB diagnosis due to its simplicity, rapid turnaround time, high sensitivity, and low cost.

## Introduction

Tuberculosis (TB) is the second most prevalent infectious disease in China with 866,000 new cases reported in 2019 [1]. Identification of acid-fast bacilli from clinical specimen remains the most reliable method for diagnosing TB. Globally in 2019, 43% of all pulmonary TB (PTB) cases were diagnosed without bacteriological evidence (i.e. acid-fast bacilli negative) [1]. According to the 2020 Global Tuberculosis Report of the World Health Organization (WHO), the mortality rate of smear-negative PTB could reach 20%, which might largely be attributed to delayed diagnosis. Smear-negative cases are also responsible for up to 20% of TB transmission at the community level [2]. With this in mind, developing a diagnostic method that is highly sensitive is urgently needed to control the spread of TB [3].

Xpert MTB/RIF (Cepheid, USA) is an automated, integrated, and cartridge-based system that uses the GeneXpert instrument [4]. This system achieves rapid detection of both *Mycobacterium tuberculosis* (MTB) and rifampin resistance at the same time. The Xpert assay has been widely used and has changed the algorithm of TB diagnosis [5]. However, the high cost of the equipment and cartridge prevent its use by the general public, particularly in less-developed countries. The key to meeting WHO’s goal to end TB in 2035 is to develop a highly sensitive, accurate, and affordable diagnostic tool with a rapid turnaround time that can be used in resource-limited settings.

**Figure 1.**
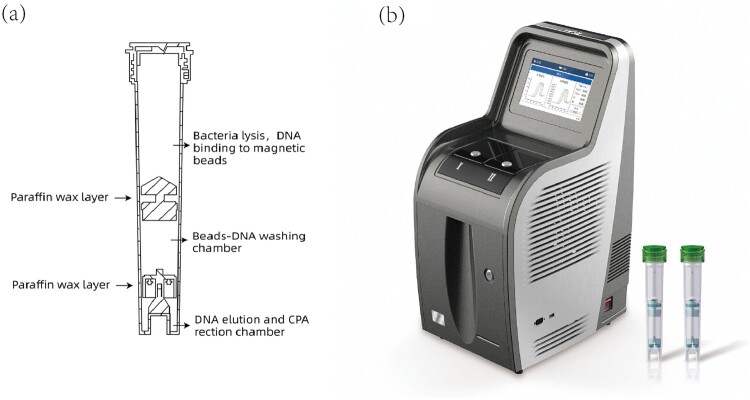
Cutaway view of the cartridge (sketch map) and equipment.

Loop-mediated isothermal amplification (LAMP) is an affordable molecular test, because a water bath could replace the expensive thermal-cycler for the nucleic acid amplification reaction [6]. Many methods based on LAMP have been developed for MTB detection [7–9] and, indeed, WHO endorsed this technique for TB diagnosis in 2016. Cross-priming amplification (CPA) is a novel LAMP method designed using specific primers to increase sensitivity while also preserving specificity. Based on this technique, the EasyNAT TB Isothermal Amplification Diagnostic Kit (short form: EasyNAT TB IAD; Ustar, Biotechnologies Co. Ltd, China) targeting the *gyrB* gene has been developed as a TB diagnosis tool [10,11]. However, EasyNAT TB IAD requires many manual processing steps, which limits its use in clinical laboratories with heavy workloads. To solve this problem, Ustar Biotechnologies developed the EasyNAT MTC assay, which is also based on CPA but targets insertion sequence Is6110. The assay uses preloaded reagents in a single cartridge that accommodates DNA extraction, DNA purification, and target gene amplification and detection using three separate chambers within the same cartridge (Figure 1). FAM dye-labeled probes were used to detect the amplification products. Since equipment compatible with EasyNAT has also been developed, the total cost is less than half that of the Xpert assay, and the whole procedure takes less than two hours. Furthermore, this second-generation test uses glassified enzyme so that the cartridge can be conveniently stored and transported at room temperature. We aimed to assess the accuracy of this novel isothermal amplification method in a clinical setting.

## Materials and methods

### Study design

From 2019 January to 2020 January, sputum samples with a minimal volume of 5 mL were prospectively and continuously collected from suspected TB patients. All these patients had symptoms suggestive of TB, including, but not limited to, long-lasting fever, persistent cough, night sweats and weight loss. In addition, abnormal radiological chest imaging was also presented. The sputum was subjected to smear testing, Lowenstein-Jensen (LJ) solid culture medium, mycobacteria growth indicator tube (MGIT) 960 culture, the Xpert MTB/RIF (Cepheid, USA) assay and EasyNAT MTC assay testing. The study was approved by the Ethics Committee of the Beijing Chest Hospital, Capital Medical University. Since all the samples used were leftover specimens from routine clinical examinations, written informed consent of the patients was waived.

### Patient diagnosis and categorization

The enrolled patients were diagnosed according to the composite reference standard (CRS), which comprises clinical findings, laboratory outcomes, radiological imaging, and follow-up data. Patient categories were defined according to the following criteria: (1) Confirmed TB: smear-positive and/or culture-positive, i.e. MTB was identified. Some patients initially had negative smear and culture outcomes in this study, but their succeeding examinations produced positive bacterial evidence. Therefore, these patients were also categorized as confirmed PTB patients but were analyzed separately. (2) Probable TB: no bacteriological evidence of TB was acquired and the PTB diagnosis was based only on symptoms, radiological images, treatment response, and follow-up data. (3) Non-TB: cancer or other diseases diagnosed by histopathological examination or other tests.

### Smear and culture

A direct smear was prepared and stained with auramine, and then examined by light-emitting diode microscopy. A 2 mL sample was decontaminated with 2–4 mL N-acetyl-L-cysteine-2% sodium hydroxide (BBL MycoPrep; Becton Dickinson, Sparks, MD) for 20 min, neutralized with sterile saline phosphate buffer (PBS; pH 6.8) to a final volume of 45 mL, and then centrifuged at 3,000 rpm for 15 min at 4°C. The pellet was re-suspended in 1.5 mL of PBS; 0.5 mL was inoculated into the MGIT 960 system (Becton, Dickinson and Company, USA) and 0.5 mL was inoculated into the LJ solid medium. All positive cultures were tested with MPT64 antigen to confirm the presence of MTB (HANGZHOU GENESIS BIODETECTION AND BIOCONTROL CO., LTD, China).

### Xpert MTB/RIF assay

The Xpert assay was performed as per the manufacturer’s instructions. Raw sputum specimens of 1-2 mL were mixed with 2 volumes of the Xpert sample processing reagent, mixed by vortex for at least 10 s, and then incubated at room temperature for 10 min. Then the specimens were mixed by vortex for 10 s and incubated at room temperature for another 5 min. A total of 2 mL of the mixture was transferred into the Xpert cartridge and loaded into the GeneXpert instrument. The automatic detection procedure was then run.

### EasyNAT MTC assay

Specimens of 1–2 mL were mixed with either 2 or 4 volumes of 4% sodium hydroxide solution depending on the viscosity of the sputum, then vortex-mixed for 30 s and allowed to settle for 15 min at room temperature until fully liquefied. One mL of liquefied sputum was transferred into a 1.5 mL Eppendorf tube and centrifuged at 12,000 rpm for 3 min. The pellet was suspended with DNA extraction liquid premixed with the internal control, then transferred into the reaction cartridge and placed in the isothermal equipment. DNA extraction, DNA purification, target gene amplification, and target gene detection can be performed in the cartridge within 90 min.

### Data management

SPSS version 19.0 (IBM, Armonk, NY, USA) was used to compare the baseline clinical characteristics and the demographic data via Mann–Whitney U test for continuous variables and Chi-square test for categorical variables. The sensitivity, specificity, positive predictive values (PPV), and negative predictive values (NPV) of the EasyNAT assay were calculated against the CRS with the following URL: http://vassarstats.net.

## Results

### Patient characteristics

**Figure 2.**
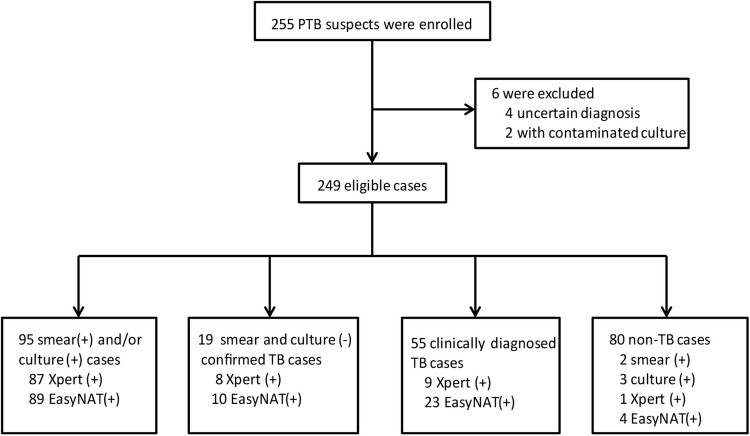
Recruitment and diagnostic classification of the participants.

A total of 255 suspected TB patients were recruited. Six participants were excluded from the study because of an uncertain diagnosis or failed testing. The final sample size for the study was 249 patients, of whom 169 (67.87%) were diagnosed with PTB (Figure 2). A total of 114 patients were categorized as confirmed PTB cases, including 19 with subsequently acquired bacterial evidence, while another 55 were categorized as probable PTB cases according to CRS. The other 80 (32.13%) cases were classified as non-TB patients, including 42 lung cancer patients and 38 patients with other infectious diseases, of which five were caused by non-tuberculous mycobacteria (NTM). Among these five patients, three were culture-positive and two of them were smear-positive, while another two were diagnosed by molecular testing with biopsy tissues. These five patients, including three *M. intracellulare*, one *M. kansasii* and one *M. abscessus*, were classified as non-TB patients for analysis in this study. All the patients were HIV-negative, and the TB patients were generally older than the non-TB patients (*P *< 0.05). The characteristics of the recruited patients are shown in Table 1.

**Table 1.** Demographic and clinical characteristics of the study participants.

Characteristics	PTB patient n = 169	Non-TB patient n = 80	*P* value
Age, median (range), yr	54 (12-93)	47 (21-85)	**0**.**002**
Male	127 (75.15%)	55 (68.75%)	0.288
Treatment status			
New case	74 (43.79%)	/	
Retreated case	95 (56.21%)	/	
History of tuberculosis	34 (20.12%)	11 (13.75%)	0.223
Combined extra-pulmonary TB	42 (24.85%)	/	
Pleural TB	29 (17.16%)		
Lymphatic TB	5 (2.96%)		
TB meningitis	3 (1.78%)		
Other sites	5 (2.96%)		
Underlying disease			
Diabetes mellitus	44 (26.04%)	12 (15.00%)	0.051
Chronic kidney disease	8 (4.73%)	3 (3.75%)	0.982
Autoimmune disease	2 (1.18%)	0	1.000
HIV-negative	169 (100%)	80 (100%)	/

“/”: not applicable.

### Performance of Xpert and EasyNAT

**Figure 3.**
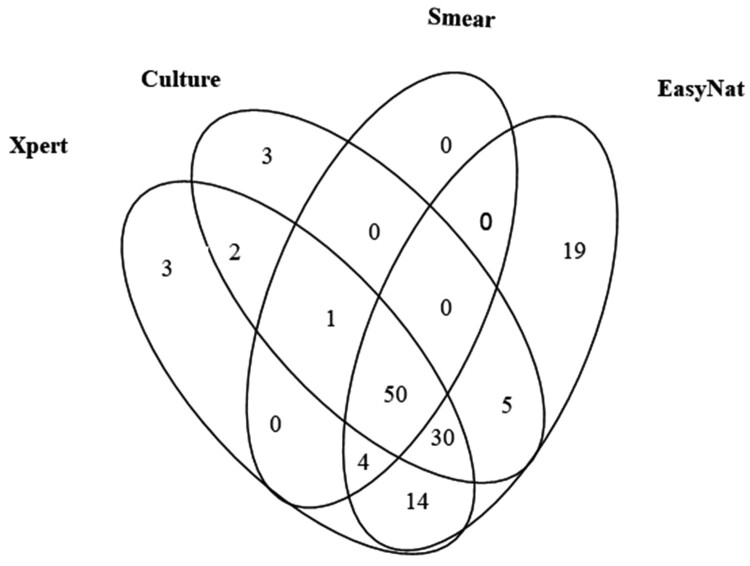
Venn diagram of the overlap between diagnostics.

The detection outcomes for all the methods for the 169 PTB patients are presented in Table 2. EasyNAT demonstrated the highest sensitivity as compared to smear, culture, and Xpert (72.19% vs. 32.54%, 53.85%, and 61.54% respectively, *P *< 0.001, χ^2 ^= 53.244; *P* < 0.001, χ^2 ^= 12.200; *P *< 0.05, χ^2 ^= 4.326). Of the 91 patients with culture-positive outcomes by any culture type, the sensitivities of smear, Xpert, and EasyNAT were 56.04% (51/91), 91.21% (83/91), and 93.40% (85/91) respectively. EasyNAT was a bit more sensitive than Xpert in the culture-positive samples but the difference was not significant (*P *= 0.578, χ^2 ^= 0.310). For the smear-positive cases, the Xpert assay detected all positive samples while EasyNAT missed one sample (Table 2, Table 3). A total of 40 patients had smear-negative but culture-positive results by at least one type of culture method. Among them, EasyNAT and Xpert detected 32 (80.00%) and 35 (87.50%) respectively. EasyNAT detected 94.23% (98/104) of Xpert positive cases, whereas Xpert detected only 80.33% (98/122) of EasyNAT positive cases. This difference was determined to be significant (χ^2 ^= 65.434, *P *< 0.0001). Of the 169 PTB cases, samples that were positive only by smear, culture, Xpert or EasyNAT were 0, 3, 3, and 19 cases respectively (Figure 3).

**Table 2.** The outcomes of different methods in testing of the 169 PTB patients.

Patient types	Case number	Positive number (%)				
		Smear	LJ	MGIT960	Xpert	EasyNAT
PTB	169	55 (32.54)	80 (47.34)	86 (50.89)	104 (61.54)	122 (72.19)
Confirmed	114	55 (48.25)	80 (70.18)	86 (75.44)	95 (83.33)	99 (86.84)
Probable	55	/	/	/	9 (16.36)	23 (41.81)
Smear+	55	/	48 (87.27)	50 (90.91)	55 (100.00)	54 (98.18)
LJ+	80	48 (60.00)	/	75 (93.75)	74 (92.50)	77 (96.25)
MGIT960+	86	50 (58.14)	75 (87.21)	/	79 (91.86)	81 (94.19)
Xpert+	104	55 (52.88)	74 (71.15)	79 (75.96)	/	98 (94.23)
EasyNAT+	122	54 (44.26)	77 (63.11)	81 (66.39)	98 (80.33)	/

“+” indicates “positive”; LJ indicates “LJ solid culture”; MGIT960 indicates “MGIT960 liquid culture”; “/” indicates not applicable.

**Table 3.** The detection outcomes of different methods for 169 PTB patients.

Case No.	Outcomes of testing			
	AFB	LJ/MGIT960	Xpert	EasyNAT
**50**	**+**	**+**	**+**	**+**
**1**	**+**	**+**	**+**	**-**
**4**	**+**	**-**	**+**	**+**
**38**	**-**	**-**	**-**	**-**
**19**	**-**	**-**	**-**	**+**
**14**	**-**	**-**	**+**	**+**
**3**	**-**	**-**	**+**	**-**
**3**	**-**	**+**	**-**	**-**
**5**	**-**	**+**	**-**	**+**
**30**	**-**	**+**	**+**	**+**
**2**	**-**	**+**	**+**	**-**

“+” indicates “positive”; LJ indicates “LJ solid culture”; MGIT960 indicates “MGIT960 liquid culture.”

Of the 74 PTB cases who had smear-negative and culture-negative results, including the 19 patients for whom bacteriological evidence was produced subsequently, EasyNAT detected more positive cases compared to Xpert [44.59% (33/74) vs. 22.97% (17/74), *P* < 0.01, χ^2^ = 7.732]. Of the 19 confirmed PTB patients, eight were Xpert positive and ten were EasyNAT positive. Among the 55 probable PTB patients who had negative smear and culture outcomes, EasyNAT yielded a much higher positive detection rate compared to Xpert [41.82% (23/55) vs. 16.36% (9/55), *P* < 0.01, χ^2 ^= 8.638].

EasyNAT was observed to have a lower specificity than the Xpert assay [95.00% (76/80) vs. 98.75% (79/80)], but the difference was not significant (*P *= 0.363, χ^2 ^= 0.862). Three lung-cancer patients and one patient infected with *M. intracellulare* produced positive results with EasyNAT, while two lung cancer patients produced positive results with Xpert.

### Positive predictive value and negative predictive value of the applied diagnostics

Among the 249 enrolled patients, the PPV for smear, LJ solid culture, MGIT960 liquid culture, Xpert, and EasyNAT were 96.49% (55/57) [95% CI: 86.84%–99.39%], 96.39% (80/83) [95% CI: 89.07%–99.06%], 96.63% (86/89) [95% CI: 89.77%–99.13%], 99.05% (104/105) [95% CI: 94.04%–99.95%], and 96.83% (122/126) [95% CI: 91.58%–98.98%] respectively. No significant difference was observed between any two methods (data not shown). The NPV for the smear, LJ solid culture, MGIT960 liquid culture, Xpert assay and EasyNAT assay testing were 40.63% (78/192) [95% CI: 33.68%–47.95%], 46.39% (77/166) [95% CI: 38.68%–54.27%], 48.13% (77/160) [95% CI: 40.21%–56.13%], 54.86% (79/144) [95% CI: 46.37%–63.09], and 61.79% (76/123) [95% CI: 52.56%–70.27%] respectively. EasyNAT demonstrated the highest NPV as compared to smear, LJ solid culture, MGIT960 liquid culture and Xpert (61.79% vs. 40.63%, 46.39%, 48.13%, 54.86% respectively. *P *< 0.001, χ^2 ^= 13.438; *P *< 0.01, χ^2 ^= 6.728; *P *< 0.05, χ^2 ^= 5.228; *P *= 0.253, χ^2 ^= 1.307)

## Discussion

This study aimed to assess the accuracy and feasibility of the EasyNAT MTC assay. EasyNAT is a modified LAMP test that is affordable and has a rapid turnaround time. After a few manual processing steps with the sputum sample, DNA extraction, amplification, and detection are performed automatically within 90 min within a single cartridge. In contrast to Xpert assay, EasyNAT requires the centrifugation of sputum, which makes it less convenient. Another advantage of the Xpert assay over EasyNAT is that rifampin resistance can be detected simultaneously. Additionally, the EasyNAT platform can hold only two cartridges at once, therefore the throughput of the equipment definitely needs be expanded in the future.

In this study, EasyNAT had generally higher positive rates than Xpert (72.19% vs. 61.53%, *P *< 0.05, χ^2 ^= 4.326). Although sensitivities of both assays for culture-positive PTB patients were not significantly different, there was a significant difference noted when testing the culture negative PTB patients. The EasyNAT assay also demonstrated better performances than the Xpert assay in the smear-negative-culture-positive PTB group and the probable PTB group. Apart from missing one smear-positive case, the EasyNAT assay detected more PTB cases compared to the Xpert assay in all the stratified analyses of the enrolled PTB patients.

EasyNAT, as the second-generation CPA, is supposed to be more sensitive than the first-generation EasyNAT TB IAD due to the switch in target gene from *gyrB* to IS6110. The rationale of this design is that the genome of MTB has multiple copies of IS6110 but only a single copy of *gyrB*. This alteration could be the explanation for the higher sensitivity of the EasyNAT rather than the Xpert assay, which uses the *rpoB* gene that has a single copy in the genome as the target gene. The results of EasyNAT in this study are similar to the reported outcomes of EasyNAT TB IAD by Fang et al [10] but are better than those by Ou et al [11,12]. In two multiple-centered evaluation studies performed by Ou et al [11,12], EasyNAT TB IAD and another LAMP kit named RalAmp (DEAOU Biotech Co., Ltd., Guangzhou, China) yielded 59.80% and 60.08% sensitivity among smear-negative and LJ solid culture–positive PTB cases respectively. Whereas EasyNAT TMC and GeneXpert obtained much higher sensitivities in our study, 90.63% (29/32) and 81.25 (26/32) respectively. Nevertheless, all the four diagnostic methods produced about 100% sensitivities among smear-positive and LJ solid culture–positive PTB cases (data not shown). The sensitivity discrepancies between these kits might be attributed to the target gene change, automation scale of operation and the quality of the kit. A previous study showed that the sensitivity of EasyNAT TB IAD and Xpert MTB/RIF in the culture positive fine-needle aspiration biopsy of tuberculosis lymphadenitis in children was 22.22% (2/9) and 55.56% (5/9) respectively. Although the sensitivity of Xpert MTB/RIF was obviously greater than that of EasyNAT TB IAD, comparison was limited by the very small sample size [13]. However, since the strategies of patient enrollment in these studies were different, these comparisons might not be very meaningful.

The EasyNAT assay was more sensitive than Xpert MTB/RIF assay among PTB patients in this study, whereas the lower specificity raised concern about the authenticity of the positive results. Due to the very small number of false positive cases, further analysis was impossible in this study. The second generation of Xpert, named Xpert-Ultra, has been developed to specifically increase sensitivity [14]. It uses the same GeneXpert platform but two extra target sequences (IS6110/IS1081) were added. Xpert-Ultra demonstrated increased sensitivity in sputum and other specimen types such as pleural fluid, CSF, and pus for extra-pulmonary TB [15–17]. However, Xpert-Ultra has been observed to have a lower specificity in sputum samples as compared to Xpert. A previous history of TB was considered to be the reason for the decreased specificity, which we presume could also affect EasyNAT’s specificity. Given this information, we assume that the EasyNAT assay might have a higher specificity in areas where the prevalence of TB is low, as there would be fewer people with a history of TB. Xpert-Ultra showed higher sensitivity but comparable specificity in comparison with the Xpert assay for extra-pulmonary TB diagnosis. Whether the EasyNAT assay also harbors the same characteristic is worthy of investigation. Since Xpert-Ultra has not been approved by the Chinese FDA for clinical use up to now, we did not use it as a control in this study. A comparison of EasyNAT and Xpert-Ultra would be worthwhile in the future to investigate their performances and cost-effectiveness.

Based on the features of the EasyNAT assay, we speculate that EasyNAT could be used as an initial test for diagnosing TB. Its high sensitivity, rapid turnaround time, and lower cost make EasyNAT a practical yet effective choice in low and middleincome countries for point-of-case testing. However, further validations in different settings are needed to justify this assumption.
